# The impact of facility type on surgical outcomes in colon cancer patients: analysis of the national cancer database

**DOI:** 10.1007/s00464-024-11230-x

**Published:** 2024-09-13

**Authors:** Ashley Shustak, Nir Horesh, Sameh Hany Emile, Zoe Garoufalia, Rachel Gefen, Ebram Salama, Stephen Sharp, Steven D. Wexner

**Affiliations:** 1 Department of Colorectal Surgery, Ellen Leifer Shulman and Steven Shulman Digestive Disease Center, Cleveland Clinic Florida, 2950 Cleveland Clinic Blvd., Weston, FL 33331 USA; 2 Department of Surgery and Transplantations, Affiliated with the Faculty of Medicine, Sheba Medical Center, Ramat Gan, Tel Aviv University, Ramat Gan, Israel; 3 Colorectal Surgery Unit, Faculty of Medicine, Mansoura University, Mansoura, Egypt; 4 Department of General Surgery, Hadassah Medical Organization and Faculty of Medicine, Hebrew University of Jerusalem, Jerusalem, Israel; 5 Department of Colorectal Surgery, Virginia Commonwealth University, Richmond, VA USA

**Keywords:** Colon cancer, Facility type, Surgical outcomes

## Abstract

**Background:**

The type of facility where patients with colon cancer are treated may play a significant role in their outcomes. We aimed to investigate the influence of facility types included in the National Cancer Database (NCDB) on surgical outcomes of colon cancer.

**Methods:**

Retrospective cohort analysis of all patients with stage I–III colon cancer included in the NCDB database between 2010 and 2019 was performed. Patients were grouped based on facility type: Academic/Research Programs (ARP), Community Cancer Programs (CCP), Comprehensive Community Cancer Programs (CCCP), and Integrated Network Cancer Programs (INCP). Study outcomes included overall survival, 30- and 90-day mortality, 30-day readmission and conversion to open surgery.

**Results:**

125,935 patients were included with a median age of 68.7 years (50.5% females). Most tumors were in the right colon (50.6%). Patient were distributed among facility types as ARP (n = 34,321, 27%), CCP (n = 12,692, 10%), CCCP (n = 54,356, 43%), and INCP (n = 24,566, 19%). In terms of surgical approach, laparoscopy was more commonly used in ARP (46%) (p < 0.001). Laparotomy was more common in CCP (58.7%) (p < 0.001), and conversely, CCP had the least amount of robotic surgery (3.9%) (p < 0.001). Median overall survival was highest in ARP (129 months, 95% CI 127.4–134.1) and lowest in CCP (103.7 months, 95% CI 100.1–106.7) (p < 0.001). Conversion rates were comparable between ARP (12%), CCCP (12%) and INCP (11.8%) but were higher in CCP (15.5%) (p < 0.001). 30-day readmission rates and 30-day mortality rates were significantly lower in ARP compared to other facility types (p < 0.001).

**Conclusion:**

Our findings display differences in surgical outcomes of colon cancer patients among facility types. The findings suggest better outcomes in terms of operative access and survival at ARP as compared to other facilities. These findings underscore the importance of understanding facility-specific factors that may influence patient outcomes and can guide resource allocation and targeted interventions for improving colon cancer care.

Colorectal cancer is the second most common cause of cancer-related death in the United States [1]. It is estimated that over 150,000 patients will be diagnosed with colon cancer in 2024. The proportion of colon cancer diagnosed amongst younger individuals is on the rise in recent years [2]. Surgery remains the gold standard treatment and the only curative option for many stages of colon cancer [3].

The US healthcare system is highly variable and there are many types of treatment centers across the country. Some are community based and others are cancer specific and known nationally. As a result, patients treated for cancer, and colon cancer in particular, are treated in different types of treatment facilities, according to a number of factors, including their insurance coverage, area of residence and access to healthcare. In an attempt to standardize cancer treatment across the country, the Committee on Cancer (CoC), which was established more than a century ago, gave certain facilities accreditation for cancer care, with over 1500 centers included in their registry today. These facilities receive their cancer program category from the American College of Surgeons based on the number of cancer diagnoses per year. Academic/Research Programs (ARP) participate in postgraduate medical education and access more than 500 new diagnoses of cancer each year. Community Cancer Programs (CCP) access more than 100 but fewer than 500 newly diagnosed cancers each year. Comprehensive Community Cancer Programs (CCCP) access 500 or more newly diagnosed cancer cases each year and Integrated Network Cancer Programs (INCP) belong to an organization that offers integrated cancer care services in a group of facilities [4].

Surgical outcomes based on the type of treatment facility have been studied in different settings in the literature [5]. At the Veterans Administration Hospitals, teaching hospitals had higher complication rates, but overall mortality rates and length of stay did not differ to non teaching hospitals within the Veterans Administration Health System [6]. In addition, cancer treatment outcomes have shown improvement in facilities with higher patient volumes [7]. Specifically, cancer margin status and survival between facility types has been studied showing decreased mortality and improved overall survival [8–10]. Despite this, evidence showing the relationship between facility type and surgical outcomes for colon cancer has been limited [11].

We assessed the characteristics, surgical treatment and outcomes of colon cancer patients undergoing surgery over a 10-year period based on the facility type in which colon cancer patients were treated using the U.S. National Cancer Database (NCDB). Our goal is to shed some light on discrepancies between these treatment centers and the outcomes of patients treated within them.

## Materials and methods

### Study design

A retrospective analysis of patients with colon cancer was performed using the NCDB (2010–2019) database. Institutional Review Board (IRB) approval was not necessary for this study as it was a retrospective review of a public data set with deidentified patient data. The NCDB compiles information from over 1500 hospitals accredited by the CoC in the United States. It is a collaborative initiative between the American College of Surgeons’ CoC and the American Cancer Society. The aim of our study was to determine if surgical outcomes for patients with colon cancer differed based on the type of facility where they underwent surgery.

### Study population

**Fig. 1:**
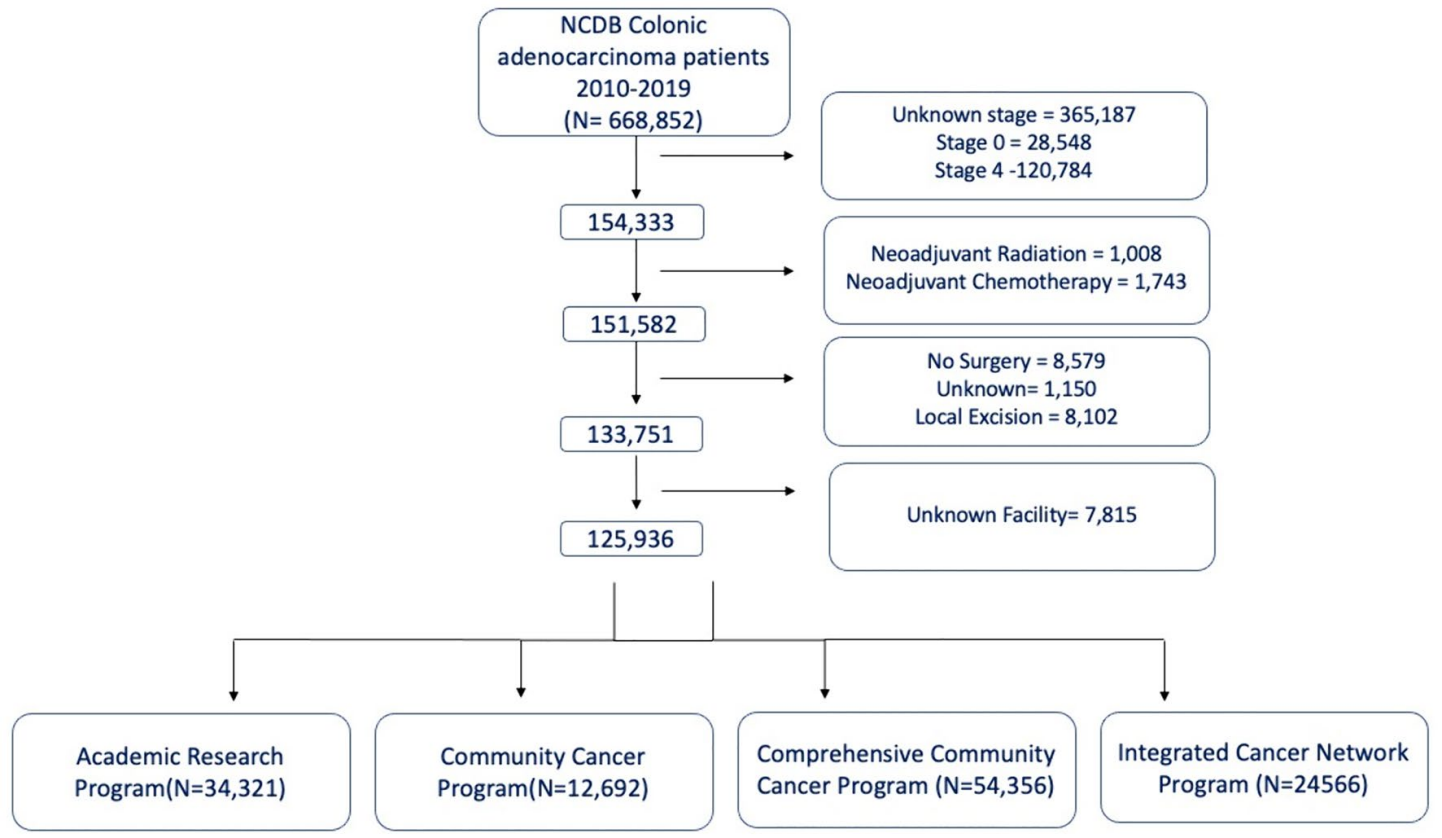
Study flow chart

The inclusion criteria included patients diagnosed with colonic adenocarcinoma, mucinous adenocarcinoma and signet-ring cell carcinoma (*International Classification of Diseases for Oncology, Third Edition* [*ICD-O-3*] codes 8140/3, 8480–8481/3, 8490/3) registered in the NCDB between 2010 and 2019 with clinical stage I–III cancers (Fig. 1).

The exclusion criteria included patients with unknown clinical staging, stage 0 and metastatic patients (stage 4). In addition, we excluded patients treated with neoadjuvant radiation and neoadjuvant chemotherapy, patients who did not undergo surgery, who underwent an unknown surgery and patients who underwent local excision as their definitive surgical procedure. Finally, we excluded patients without data on the type of facility in which they were treated.

### Data collection

The following patient data were collected and used for the analysis: age; sex; race and ethnicity; Charlson Comorbidity Index (CCI) score; clinical and pathologic TNM (tumor, node, metastasis) stages; insurance status; geographic region; tumor histology and grade; tumor location; pathologic stage; type and approach of surgery; and days from diagnosis to surgery. Primary outcomes include overall survival, 30- and 90-day mortality, 30-day readmission and conversion to open surgery, secondary outcomes include surgical margins, examined and positive regional nodes, and length of inpatient hospital stay (days). Positive surgical margins included both circumferential and radial margins and entailed both microscopic and macroscopic tumor identification.

### Statistical analysis

EZR, version 1.55,14 R software, version 4.1.2 (The R Foundation), and SPSS, version 23 (IBM), were employed for statistical analyses [12]. Continuous data were presented as mean and standard deviation (SD) for normally distributed variables, or as median and interquartile range (IQR) for non-normally distributed values. Unpaired, two-tailed t-tests or one-way ANOVA tests were utilized to assess continuous variables. Categorical data, expressed in numbers and percentages, underwent analysis through Fisher’s exact test or the χ^2^ test. To handle missing data in the primary outcomes, a complete case analysis approach was implemented. The statistical significance threshold was set at a two-sided p-value of < 0.05.

## Results

### Description of the cohort

A total of 125,935 patients diagnosed with colonic adenocarcinoma were included in the analysis. 50.5% of patients were female with a mean age of 68.9 (SD 12.34) years. Most tumors were located in the right colon (50.6%), followed by the left colon (36.5%) the transverse colon (10.2%), and non-specified lesions (3%). The majority of patients had a histology of adenocarcinoma (89.3%), whereas mucinous adenocarcinoma represented (9.5%). Most of the patients had a clinical stage 1 disease (47.2%) followed by patients diagnosed with stage 2 disease (32.3%).

### Analysis based on the facility type

Differences in demographic, clinical and treatment characteristics among patients treated in the different facility types can be seen in Table 1. The most common facilities in which patients were treated for colon cancer were CCCPs (n = 54,356, 43.2%), followed by ARP (n = 34,321, 27.3%), INCP (n = 24,566, 19.5%), and finally CCPs (n = 12,692, 10.1%). Patients’ sex and median age were comparable between the different facility types. Most patients were insured through Medicare throughout all types of facilities, however the rate of patients with private insurance was higher at ARPs (35.8%, p < 0.001).

**Table 1:** Comparison of patient demographic, clinical and surgical factors between patients who underwent surgery at different facility types

Factor	Academic/research program (n = 34,321, 27.3%)	Community cancer program (n = 12,692, 10.1%)	Comprehensive community cancer program (n = 54,356, 43.2%)	Integrated network cancer program (n = 24,566, 19.5%)	p value
Mean age–years (SD)	67.22 (12.41)	69.90 (12.19)	69.52 (12.25)	69.46 (12.34)	< 0.001
Sex (%)					0.016
Female	17,355 (50.6)	6566 (51.7)	27,840 (51.2)	12,721 (51.8)	
Male	16,966 (49.4)	6126 (48.3)	26,516 (48.8)	11,845 (48.2)	
Race (%)					< 0.001
White	26,196 (77.1)	11,042 (87.6)	46,840 (86.7)	20,693 (84.9)	
Black	5686 (16.7)	1017 (8.1)	5271 (9.8)	2820 (11.6)	
Asian	1466 (4.3)	385 (3.1)	1357 (2.5)	592 (2.4)	
American Indian	89 (0.3)	61 (0.5)	199 (0.4)	63 (0.3)	
Other	534 (1.6)	96 (0.8)	386 (0.7)	216 (0.9)	
Geographic classification					< 0.001
Metro	30,067 (90.9)	8704 (70.0)	44,963 (84.1)	21,426 (89.1)	
Rural	299 (0.9)	306 (2.5)	1112 (2.1)	281 (1.2)	
Urban	2708 (8.2)	3428 (27.6)	7389 (13.8)	2338 (9.7)	
Charlson score (%)					< 0.001
0	24,171 (70.4)	8596 (67.7)	36,513 (67.2)	16,301 (66.4)	
1	6850 (20.0)	2851 (22.5)	12,126 (22.3)	5402 (22.0)	
2	2065 (6.0)	811 (6.4)	3733 (6.9)	1799 (7.3)	
3	1235 (3.6)	434 (3.4)	1984 (3.7)	1064 (4.3)	
Type of insurance (%)					< 0.001
Medicaid	2263 (6.8)	681 (5.4)	2034 (3.8)	1104 (4.6)	
Medicare	17,650 (52.9)	8021 (64.0)	32,817 (61.1)	14,738 (60.8)	
Private	11,939 (35.8)	3418 (27.3)	17,069 (31.8)	7629 (31.5)	
Other	245 (0.7)	127 (1.0)	466 (0.9)	238 (1.0)	
Not Insured	1275 (3.8)	285 (2.3)	1318 (2.5)	540 (2.2)	
Grade (%)					< 0.001
Well differentiated	3562 (11.0)	1774 (14.7)	6052 (11.7)	2962 (12.7)	
Moderately differentiated	22,652 (70.2)	8263 (68.7)	35,834 (69.1)	16,134 (69.2)	
Poorly differentiated	5361 (16.6)	1744 (14.5)	8771 (16.9)	3675 (15.8)	
Undifferentiated	695 (2.2)	253 (2.1)	1164 (2.2)	536 (2.3)	
Histology (%)					< 0.001
Adenocarcinoma	30,364 (88.5)	11,393 (89.8)	48,864 (89.9)	21,891 (89.1)	
Mucinous adenocarcinoma	3523 (10.3)	1165 (9.2)	4892 (9.0)	2384 (9.7)	
Signet-ring cell carcinoma	434 (1.3)	134 (1.1)	600 (1.1)	291 (1.2)	
Tumor location (%)					< 0.001
Left colon	12,517 (36.5)	4480 (35.3)	18,861 (34.7)	8757 (35.6)	
Right colon	17,364 (50.6)	6406 (50.5)	28,291 (52.0)	12,656 (51.5)	
Transverse colon	3447 (10.0)	1294 (10.2)	5808 (10.7)	2501 (10.2)	
Not specified	993 (2.9)	512 (4.0)	1396 (2.6)	652 (2.7)	
TNM stage group (%)					< 0.001
I	16,422 (47.8)	5571 (43.9)	25,409 (46.7)	12,035 (49.0)	
II	10,840 (31.6)	4280 (33.7)	17,844 (32.8)	7770 (31.6)	
III	7059 (20.6)	2841 (22.4)	11,103 (20.4)	4761 (19.4)	
Surgery type					< 0.001
Hemicolectomy	30,528 (88.9)	11,625 (91.6)	49,702 (91.4)	22,177 (90.3)	
Hemicolectomy+contiguous organ	2344 (6.8)	755 (5.9)	3156 (5.8)	1605 (6.5)	
Total colectomy	1212 (3.5)	248 (2.0)	1264 (2.3)	634 (2.6)	
Total colectomy+contiguous organ	237 (0.7)	64 (0.5)	234 (0.4)	150 (0.6)	
Type of Surgery (%)					< 0.001
Laparoscopic or endoscopic	15,885 (49.9)	4455 (37.3)	24,539 (47.5)	11,245 (48.1)	
Open or unspecified	13,770 (43.2)	7008 (58.7)	23,794 (46.1)	10,421 (44.5)	
Robotic assisted	2196 (6.9)	470 (3.9)	3335 (6.5)	1735 (7.4)	

*SD* standard deviation, *SMD* standardized mean difference

**Fig. 2:**
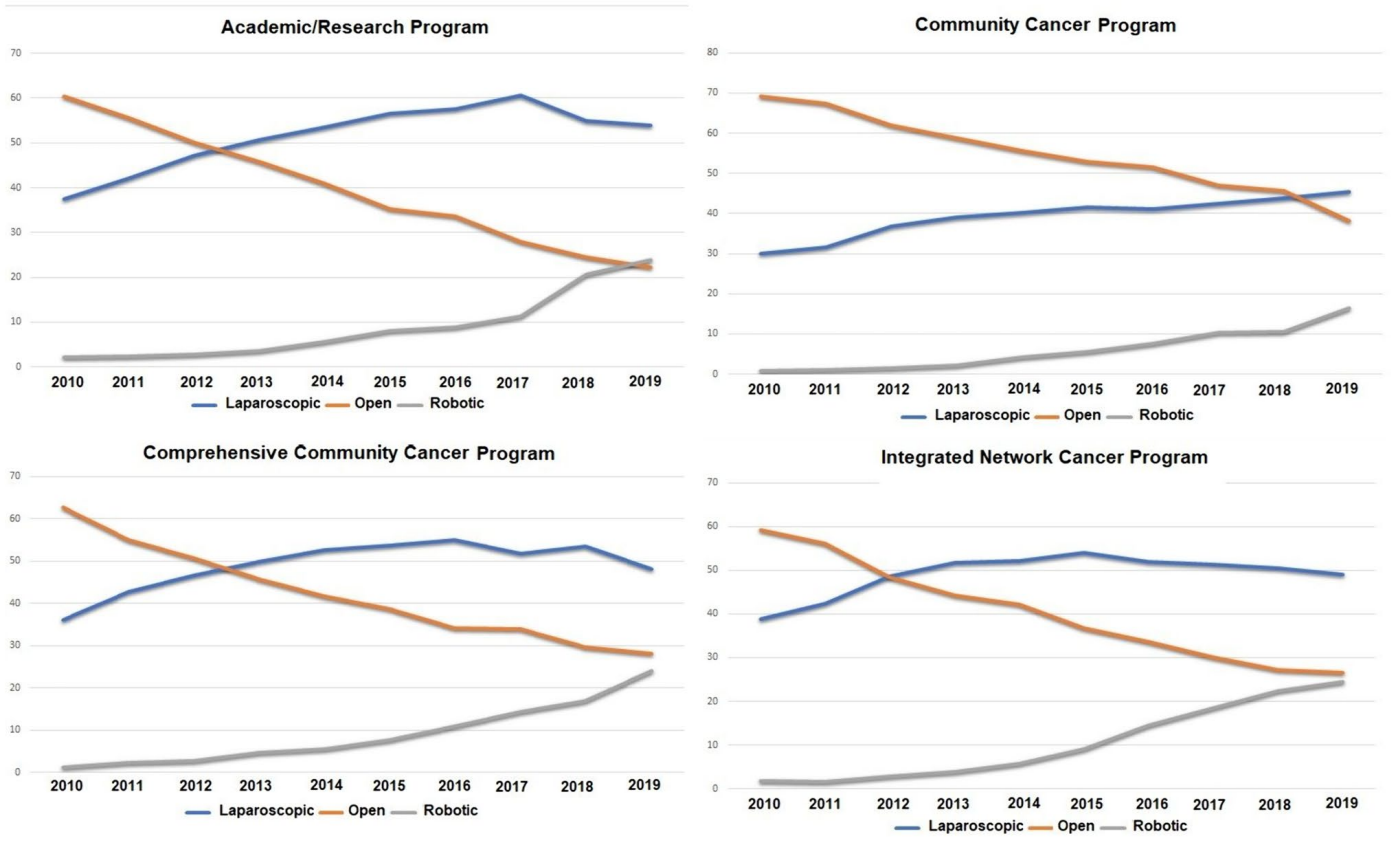
Approach to surgery across all facility types during the study years

Most tumors were located in the right colon across all facility types. Hemicolectomy was the most common procedure with a slightly higher rate of total abdominal colectomy and contiguous organ resection seen in ARP’s. In terms of surgical approach, laparoscopy was more commonly used in ARPs (49.9%), CCCPs (47.5%) and INCP’s (48.1%) compared to CCP’s (37.3%) (p < 0.001). INCPs had the highest rate of robotic procedures (7.4%). A significant increase in robotic surgery procedures was seen in all facilities across the study years, but the trend was moderate in CCP’s compared to other facility types (Fig. 2).

### Outcomes

Patients’ outcomes across the different facility types are detailed in Table 2. The rate of positive surgical margins was slightly lower in ARP’s (3.8%) compared to other centers (p < 0.001). Conversion rates were comparable between ARPs (12%), CCCPs (12%) and INCPs (11.8%) but were higher in CCPs (15.5%) (p < 0.001). 30-day (1.9%) and 90-day (3.4%) mortality rates were significantly lower in ARPs compared to other facility types (Table 2).

**Table 2:** Clinical and pathologic outcomes in patients undergoing surgery at different facility types

Factor	Academic/research program (n = 34,321)	Community cancer program (n = 12,692)	Comprehensive community cancer program (n = 54,356)	Integrated network cancer program (n = 24,566)	p-value
Surgical margins (%)					< 0.001
Negative	32,463 (96.2)	11,937 (95.1)	51,302 (95.0)	23,327 (95.6)	
Positive	1277 (3.8)	620 (4.9)	2700 (5.0)	1066 (4.4)	
Pathologic TNM stage group (%)					< 0.001
0	146 (0.4)	32 (0.3)	245 (0.5)	108 (0.5)	
I	9309 (28.5)	3396 (28.2)	14,678 (28.6)	6758 (29.2)	
II	11,062 (33.8)	4295 (35.6)	18,151 (35.4)	8190 (35.3)	
III	11,453 (35.0)	4130 (34.3)	17,178 (33.5)	7711 (33.3)	
IV	728 (2.2)	204 (1.7)	1082 (2.1)	413 (1.8)	
Conversion (%)					< 0.001
No	15,909 (88.0)	4163 (84.5)	24,529 (88.0)	11,448 (88.2)	
Yes	2172 (12.0)	762 (15.5)	3345 (12.0)	1532 (11.8)	
Median number of examined lymph nodes (IQR)	19 [0–90]	17 [0–90]	17 [0–90]	18 [0–90]	< 0.001
Median number of positive lymph nodes (IQR)	0 [0–62]	0 [0–67]	0 [0–85]	0 [0–49]	0.001
Median length of stay following surgery (IQR)	5 [0–160]	5 [0–169]	5 [0–173]	5 [0–170]	< 0.001
30-day readmission rate (%)	1551 (4.6)	557 (4.4)	2696 (5.0)	1251 (5.1)	0.001
30-day Mortality (%)	604 (1.9)	369 (3.0)	1472 (2.9)	716 (3.1)	< 0.001
90-day Mortality (%)	1098 (3.4)	613 (5.1)	2463 (4.8)	1177 (5.1)	< 0.001
Overall survival (%)	22,211 (68.5)	7472 (61.2)	31,865 (61.5)	14,660 (63.0)	< 0.001

*IQR* interquartile range

Median overall survival was highest in the ARPs (129 months, 95% CI 127.4–134.1) compared to all other facility types. INCPs (111 months, 95% CI 109.2–114.0) and CCCPs (108 months, 95% CI 106.5–110.3) had comparable overall survival and CCP’s had the lowest median survival (103.7 months, 95% CI 100.1–106.7) (p < 0.001). Univariable Cox regression analysis of overall survival found the risk for mortality was highest in CCP (HR 1.38), followed by CCCP’s (HR 1.36) and INCP’s (HR 1.28) compared to ARP.

Multivariate analysis for factors associated with overall survival demonstrated that in comparison to ARP, treatment in all other facilities was associated with a moderately increased risk for mortality, however, the increased risk was similar among CCPs (HR 1.08, 95% CI 1.05–1.13; p < 0.001), CCCP’s (HR 1.09, 95% CI 1.07–1.12; p < 0.001) and INCP’s (HR 1.09, 95% CI 1.06–1.13; p < 0.001) (Table 3). There was an 8–9% increase in the chance of mortality with patients treated at facilities other than academic research programs.

**Table 3:** Multivariate regression analysis of factors associated with overall survival

Variable	Hazard Ratio	95%CI	p-value
Age	1.05	1.04–1.06	** < 0.001**
Male sex	1.23	1.21–1.26	** < 0.001**
Race			
American Indian	1.26	1.06–1.52	**0.01**
Black	1.09	1.06–1.14	** < 0.001**
Asian	0.70	0.65–0.76	** < 0.001**
Other	0.75	0.66–0.87	** < 0.001**
Charlson score			
1	1.23	1.21–1.27	** < 0.001**
2	1.64	1.59–1.70	** < 0.001**
3	2.06	1.98–2.16	** < 0.001**
Geographic region			
Urban	1.08	1.05–1.12	** < 0.001**
Rural	1.10	1.02–1.19	0.009
Insurance status			
Medicare	0.76	0.72–0.81	** < 0.001**
Private	0.65	0.62–0.7	** < 0.001**
Other	0.67	0.59–0.78	** < 0.001**
No insurance	0.94	0.87–1.03	0.21
Facility type			
Community cancer program	1.08	1.05–1.13	** < 0.001**
Comprehensive community cancer programs	1.09	1.07–1.12	** < 0.001**
Integrated network cancer programs	1.09	1.06–1.13	** < 0.001**
Grade			
Moderately differentiated	1.07	1.04–1.11	** < 0.001**
Poorly differentiated	1.40	1.35–1.46	** < 0.001**
Undifferentiated	1.35	1.27–1.45	** < 0.001**
Histology			
Mucinous	1.09	1.06–1.13	** < 0.001**
Signet ring cell	1.54	1.43–1.67	** < 0.001**
Tumor location			
Right colon	0.95	0.93–0.98	** < 0.001**
Transverse colon	1.01	0.98–1.05	0.43
Unspecified	1.10	1.04–1.18	**0.001**
Adjuvant chemotherapy	0.88	0.87–0.91	** < 0.001**
Type of surgery			
Total colectomy	1.23	1.16–1.31	** < 0.001**
Hemicolectomy+contiguous organ	1.31	1.26–1.36	** < 0.001**
Total colectomy+contiguous organ	1.37	1.21–1.56	** < 0.001**
Surgical approach			
Laparoscopic	0.76	0.75–0.78	** < 0.001**
Robotic	0.63	0.59–0.67	** < 0.001**

Bold text in the p value column indicates statistical significance

*CI* confidence interval

## Discussion

This study aimed to determine the surgical outcomes of patients undergoing surgery for colon cancer at different types of facilities. We evaluated data from over 125,000 patients during a 10-year period using the NCDB database. Our data shows that surgical approach, outcomes and survival differ significantly among facility types. A significant number of patients are being treated at non academic or cancer specific facilities for their cancer care and the treatments they should expect to receive may be different than that at an ARPs.

Interestingly, our results showed improved survival after colon cancer surgeries at ARPs. Similar results have been seen in the literature with improved overall survival after surgery in patients with brain metastases, melanoma, and lung cancer [13–15]. Receiving treatment for brain metastases from any cancer at academic centers was associated with improved overall survival compared to patients treated at non-academic facilities [13]. Possible reasons for this could be in part due to the enhanced research, clinical trials, and stricter adherence to evidence-based practice parameters at academic facilities. Potentially patients treated at academic centers have better outcomes because they have less comorbidities or have more resources than those treated in the community. In part this may be due to the higher case volume at academic centers, though overall the highest percentage of patients in our study were treated at CCCPs. The NCDB showed that facilities with higher surgical volumes had better outcomes for lung cancer patients than those with lower volumes [9]. Similarly, this was seen for colorectal cancer patients’ 5-year survival, which was significantly improved at high-volume hospitals by high-volume surgeons [16].

Regarding surgical treatment, the database shows a trend to ARPs performing more minimally invasive surgery (MIS) while community hospitals show a slower rise. Interestingly, our data also showed that surgical approach had a significant association with overall survival, which could be either a selection bias as more challenging cases tend to be operated on via open surgery or the survival benefit can be true and attributable to better tumor clearance and less surgical trauma with MIS. It is well known that patients benefit from MIS approaches in colon surgery with less pain, faster recovery, earlier return of bowel function and shorter hospital stays [18–20]. Furthermore, studies demonstrated that a MIS approach and even conversion to a MIS approach from an open approach can have benefits for patient survival. Hakmi et al. [21] demonstrated that minimally invasive colon resections are associated with improved morbidity and mortality to open surgery. Horesh et al. [17] demonstrated that conversion to open surgery from MIS compared to upfront laparotomy in colon cancer was associated with improved outcomes.

Minimally invasive surgery techniques have been used with more frequency at hospitals with large volumes on colorectal surgery and academic centers [22, 23]. Villano et al. [22] used the NCDB database to show MIS utilization at different facility types from 2010–2015 demonstrating the community hospitals had lower rates of MIS, MIS uptake, and higher conversion rates. Our study expands on the data through 2019 and demonstrates that the open approach is still used more consistently at CCPs. Our data showed improved survival in robotic surgery compared to MIS, which is supported in the literature with elderly patients having improved survival with robotic colectomies [24]. In addition, there has been no increase in the percentage of robotic procedures despite newer data and more uptake of robotic surgery usage.

The database showed conversion rates differed among facility types with ARPs having slightly lower rates 12%. CCPs reported the highest conversion rates at 15.5%. Simorov et al. [25] utilized the University HealthSystem Consortium database to estimate the overall rate of laparoscopic conversion to open in colon cases from 2008 to 2011 is 15%. The majority of the University HealthSystem Consortium database is from academic facilities, which demonstrates NCDB has a slightly lower rate of conversion at academic facilities than previously reported in the literature. This might be because the data was newer and surgeons have become more facile with laparoscopy. Similarly, Moghadamyeghaneh et al. [26] used the NIS to demonstrate between 2009 and 2012 laparoscopic to open colorectal surgical conversions were 14.3%. More importantly, converted laparoscopic procedures have higher wound infection rates, postoperative ileus, morbidity and mortality than procedures that were completed laparoscopically [27, 28].

### Limitations

Our study has several limitations. First, large databases are prone to type 1 errors, showing significance despite minimal changes between the study groups. For that reason, we tried to focus on findings with significant clinical relevance. In addition, a large number of patients were excluded from the database due to missing details, mainly clinical staging which is key for determining survival outcome. Some of our statistically significant outcomes may not have affected the overall outcome of patients such as time to definitive surgery, where 10–15 days would not cause drastic changes in the progression of cancer or complication rate. Furthermore, important clinical data including patient’s body mass index, nutritional status and detailed comorbidities are not included in the NCDB. In addition, data on previous abdominal interventions and the experience and training of the performing surgeon were also not available. These parameters, as well as several others, might have given a more detailed explanation to our findings, including the differences in MIS rates at different hospitals.

## Conclusions

Our findings display differences in surgical outcomes of colon cancer patients among facility types. The findings suggest higher access to MIS and better survival outcome in ARP’s, CCCP’s and INCP’s compared to CCP’s. We recommend referring complex cases of colon cancer that are planned to be treated at CCPs to a more specialized center to obtain better outcomes. These findings underscore the importance of understanding facility-specific factors that may influence patient outcomes and can guide resource allocation and targeted interventions for improving colon cancer care.

## Data Availability

Upon reasonable request to first author.
